# Evaluation of differences in the performance strategies of top and bottom basketball teams utilizing rank-sum ratio comprehensive

**DOI:** 10.3389/fspor.2022.1052909

**Published:** 2022-11-25

**Authors:** Wenping Sun, ChenSoon Chee, LianYee Kok, FongPeng Lim, Shamsulariffin Samsudin

**Affiliations:** ^1^Department of Sport Studies, Faculty of Educational Studies, Universiti Putra Malaysia, Kuala Lumpur, Malaysia; ^2^Department of Sports, Nanyang Institute of Technology, Nanyang, China; ^3^Department of Sport Science, Faculty of Applied Sciences, Tunku Abdul Rahman University of Management and Technology, Kuala Lumpur, Malaysia; ^4^Department of Mathematics and Statistics, Faculty of Science, Universiti Putra Malaysia, Kuala Lumpur, Malaysia

**Keywords:** Men's Basketball World Cup, attacking performance, defensive performance, the overall attacking and defensive ability, the final ranking

## Abstract

**Introduction:**

This study aimed to explore common characteristics among top basketball teams, differentiate attacking and defensive performance between top and bottom teams, and correlate attacking and defensive performance with final competition rankings during the 2019 Men's Basketball World Cup, as well as to determine the relationship between performance indicators and the attacking and defensive performance. In addition, the study aimed to determine the attacking and defensive level of the top and bottom eight teams and find their existing problems and shortcomings, to further improve their competitive basketball strength, and also provided valid and reliable information for coaches to conduct targeted training in the future.

**Methods:**

The rank-sum ratio (RSR) was employed to evaluate the attack, defense, and overall attacking and defensive performance between the top and bottom teams during the 2019 Men's Basketball World Cup. Additionally, an independent sample *T*-test was conducted to test the difference in performance indicators of attack and defense between the top eight and bottom eight teams. Spearman Rho Correlation was conducted to determine the relationship between the attacking and defensive RSR value and the final competition ranking at the 0.05 confidence level. Pearson Correlation was employed to test the relationship between the performance indicators and the attacking and defensive RSR value at the 0.05 confidence level. According to Spearman and Pearson Correlation, the indicators which contributed most to the attacking and defensive performance, as well as the correlation between attack and defense and the final ranking, can thus be determined.

**Results:**

The results showed that the attacking performance of the top eight teams was far better than the bottom eight teams in terms of average points (*p* = 0.000), 2-point shoot percentage (*p* = 0.001), 3-point shoot percentage (*p* = 0.003), free throw percentage (*p* = 0.001), turnovers (*p* = 0.012), and assists (*p* = 0.000), and there was a significant difference (*p* < 0.05). However, second attack (*p* = 0.484), fast-break (*p* = 0.174), and offensive rebounds (*p* = 0.261) showed no significant difference between the two cohorts (*P* > 0.05), and the offensive rebounds of the bottom eight teams were better than the top eight teams. Additionally, there was a large gap between the top eight teams and the bottom eight teams in lost points (*p* = 0.001) and defensive rebounds (*p* = 0.000), with a very significant difference (*p* < 0.01). However, steals (*p* = 0.760), blocks (*p* = 0.166), and fouls (*p* = 0.686) had no significant difference between the two cohorts (*P* > 0.05). Additionally, there was a very significant difference between attack RSR (*p* = 0.000), defense RSR (*p* = 0.006), and the overall attack-defense RSR (*p* = 0.000) of the top eight and bottom eight teams (*p* < 0.01), and most top teams focused on developing both attack and defense and paid attention to improve the overall attacking and defensive ability. Moreover, there was a significant relationship between the overall attack-defense performance and assists (*p* = 0.832), rebounds (*p* = 0.762), turnovers (*p* = 0.702), 2-point shoot percentage (*p* = 0.704), defensive rebounds (*p* = 0.809), fast-break points (*p* = 0.577), blocks (*p* = 0.600), and free throw percentage (*p* = 0.575).

**Conclusions:**

This study showed that the top basketball teams focused on developing both attack and defense, and have the common characteristics of strong attack and defense. Whether it was the attack, defense, or overall attacking and defensive ability, there was a significant relationship with the final ranking. Additionally, this study showed that there were very significant differences in both attacking and defensive abilities between the top eight and bottom eight teams, as well as highlighted their respective advantages and disadvantages in attacking and defensive indicators. Besides that, this study found that performance indicators such as assists, defensive rebounds, 2P%, turnovers, FT%, fast-breaks, and blocks were the main factors that distinguish the top and bottom teams, and they had a significant relationship with overall attacking and defensive performance. The above information allows coaches and players to learn the latest developments in competitive basketball, as well as their advantages and disadvantages, to help them organize targeted training in the future.

## Introduction

Basketball has become the third most popular sport in the world (1), being played in almost every nation without exception (2). In this century, basketball matches during competitions tend to be played at a faster pace. Advancements in sports science and technology have scientifically developed the theory, tactics, and training of basketball, and improved the competition system and rules (3). Basketball development is seen in many parts of the world and is no longer a monopoly of a few nations (4). However, even though the basketball skill level among nations is narrower than before, playing strength is still uneven (5). Many national teams are weak and often lose games with large score gaps in international elite-level basketball competitions. Those teams typically come from Africa and Asia, and they often exhibit low-quality play in comparison with European and American teams (6). Among Asian basketball teams, only teams from China have reached the top eight positions during two Olympic Games in the past 20 years, while no African team has achieved similar results (7). Therefore, it seems there is extreme imbalance and polarization in the development of basketball among teams from different regions of the world. This disparity in performance has been noticed by the basketball organizing fraternity which would like to improve the level of basketball competition and expand the influence of basketball.

With the evolution of tactical and technical in basketball games, it is important for coaches, players, and researchers to learn every detail of the sport. Performance analyses in basketball are a fundamental tool, allowing stakeholders to obtain valid and reliable information on their teams and competitors (8). This analysis can be used to not only identify the most valuable players but also evaluate each player's contribution to team performance (9, 10). Performance analysis in basketball typically focuses on performance indicators and their influence on each game's outcome (11). Performance indicators are usually reflected by game-related statistics such as scores per game, rebounds, and assists (8, 12). Quantitative analysis of basketball performance through game-related statistics has been widely used among coaches, players, and researchers to measure player performance and analyze game results (10). In this sense, some game-related statistics were used to discriminant between the winning and losing teams utilizing different methods (13). For example, the discriminant analysis method has been used by Dogan et al. (14), Ergül, et al. (15), Gómez et al. (16), Lorenzo et al. (17), and García et al. (18) to determine the variables which are related to winning and losing teams. Besides that, some authors have used non-linear and machine learning techniques to analyze basketball performance (19). Of course, some studies that using the RSR comprehensive evaluation to analyze the attacking and defensive performance of the basketball team (20–26). However, most studies have shown that the performance indicators that determine the game outcome were different. Additionally, among the articles using RSR comprehensive evaluation, most were published by Chinese scholars, but most of those compared and analyzed the attacking and defensive ability between the Chinese men's basketball team and other top teams, resulting in regional limitations.

Basketball has cooperative-opposition characteristics which is an intermittent, court-based team sport, including repeated transitions between attack and defense, and frequent changes in movements (27, 28). Therefore, attack and defense are two basic forms the basketball competition, which take place in turn (24). Unlike individual sports, team activity consists of a large number of performance indicators in attack and defense (29). In this sense, scientific analysis of technical indicators that affect the game outcome has become an important task for coaches, trainers, and sports researchers (28). However, most authors using the discriminant analysis and the non-linear like those mentioned above (14–19) only indicated that certain specific performance indicators, such as scores per game, rebounds, and assists, were important factors influencing the game outcome, but they did not link the overall attacking and defensive performance with game outcomes. Besides that, the strength of each team with the development of basketball is not static, and overall regional and world basketball performance changes with improvement in training knowledge and technology. Therefore, it is necessary to analyze basketball performance during an international elite-level basketball competition like the Men's Basketball World Cup, which represents the highest level of basketball in the world (30), in order to explore the current trends in competitive basketball.

This study aimed to explore common characteristics among top basketball teams, differentiate attacking and defensive performance between top and bottom teams, and correlate attacking and defensive performance with final competition rankings during the 2019 Men's Basketball World Cup, as well as to determine the relationship between performance indicators and the attacking and defensive performance. In addition, the study aimed to determine the attacking and defensive level of the top and bottom eight teams and find their existing problems and shortcomings, to further improve their competitive basketball strength, and also provided valid and reliable information for coaches to conduct targeted training in the future. It was hypothesized that there was a significant difference in attacking and defensive performance, as well as the attacking and defensive indicators between the top and bottom teams. Moreover, the attacking and defensive performance had a significant relationship with the final competition rankings.

## Method

### Sample

There were a total of 32 teams in the 2019 Men's Basketball World Cup. In this study, the top eight teams (Spain, Argentina, France, Australia, Serbia, the Czech Republic, the United States, and Poland) and the bottom eight teams (Montenegro, South Korea, Angola, Jordan, Côte d'Ivoire, Senegal, Japan, and the Philippines) were selected as the sample. This study was conducted based on the game-related statistics of 76 matches among these 16 teams, from the first round to the finals.

### Data collection

All data of this study were collected from the basketball database of the International Basketball Federation's official website (30). By watching the video of the top eight and bottom eight teams in the 2019 Men's Basketball World Cup, the researcher recorded game data related to this research, such as points, rebounds, assists, and steals. Then the recorded data are compared with the FIBA data to verify the validity and reliability of the data. If some data are inconsistent, the researcher watched the video again to verify and ensure the accuracy of the data.

Generally, the attacking indicators included points, 2-point shots, 3-point shots, free throws, offensive rebounds, assists, and turnovers (31), while defense indicators included lost points, defensive rebounds, steals, blocks, and fouls (22, 32). However, the basketball competition has also been greatly affected by the constant changes in rules, especially the increasing number of second attacks and fast-break in the game (33). Therefore, to reflect the attacking and defensive performance more comprehensively, this study added the second attack and fast-break in the attacking indicators.

### Data analysis

#### The RSR comprehensive evaluation method

##### Basic principles

The RSR is a comprehensive evaluation method developed by Tian Fengdiao (1988). The RSR method is based on the concept that the indicators' values can be turned into a dimensionless statistical composite index, the so-called Rank-sum ratio, through the process of rank transformation (34). The validation and rationality of the RSR method have been demonstrated by Tian Fengdiao (35). The RSR comprehensive analysis method is applicable to statistics and analysis of form and measurement data (20), which can reflect the comprehensive evaluation of different measurement units and multiple indicators in a matrix of N rows and M columns by the average value of row or column order (35). It has the characteristics of large capacity and strong plasticity and has been widely used in various research fields (31).

##### Calculation method

The calculation formula of RSR: *RSR* = ∑*R*/(*M* * *N*), where R refers to the rank value of each evaluation index; ∑R refers to the rank sum value of the evaluation index; M refers to the number of evaluation index; and N refers to the number of teams. The RSR value is between 0 and 1. As for indexes that are better when they are higher, they should be coded from small to large, while indexes that are better when they are lower should be coded from large to small. When the ranks of some indexes are the same, the average of these index values is taken (36). The larger the RSR value means the higher level of teams, and vice versa (21, 31). The 5-level evaluation standard of the RSR comprehensive evaluation method was used in this study (Table 1).

**Table 1:** Rank-sum ratio (RSR) comprehensive evaluation grade standard.

**A**	**B**	**C**	**D**	**E**
≥0.8	0.60~0.79	0.40~0.59	0.20~0.39	≤ 0.19

Percentile of Rank-Sum Ratio (RSR) as suggested by Tian Fengdiao.

#### SPSS 25.0 software statistics analysis

Using the IBM SPSS software, an independent sample *t*-test was conducted to test the difference in performance indicators of attack and defense between the top eight and bottom eight teams to reflect objectively the gap among them. Additionally, Spearman Rho Correlation was conducted to determine the relationship between the attacking and defensive RSR value and the final competition ranking at 0.05 level of significance. Pearson Correlation was employed to test the relationship between the performance indicators and the attacking and defensive RSR value at a 0.05 level of significance. According to Spearman and Pearson Correlation, the indicators which contributed most to the attacking and defensive performance, as well as the correlation between attack and defense and the final ranking, can thus be determined.

## Results

### The RSR comprehensive evaluation on attacking performance between the top eight and bottom eight teams

According to the principle of RSR comprehensive evaluation, the larger the value of each evaluation indicator, the higher the rank. However, fouls and lost points in the defensive indicators and the turnovers in the attacking indicators are low-quality indicators. For convenience in statistical analysis, the low-quality indicators are inversely assigned; that is, the larger the value, the lower the rank, to achieve the consistency of the rank trend of evaluation indicators (21). Finally, the RSR value and grade of each team's attacking performance were obtained by the RSR calculation formula. The data shown in Table 2 is the average data for each team.

**Table 2:** The RSR values and grades of attacking performance for the top eight and bottom eight teams.

**FR**	**Teams**	**Points**	**R**	**2P %**	**R**	**3P %**	**R**	**FT %**	**R**	**SCP**	**R**	**FBP**	**R**	**OR**	**R**	**As**	**R**	**To**	**R**	**RSR**	**Grade**	**Rank**
1	Spain	84.4	12	55.6	14	31.7	7.5	76.5	12	10.9	15	9.8	11	11.4	11	22.8	14.5	12.4	12	0.76	B	2
2	Argentina	86.0	14	50.8	9	35.4	12	78.2	14	8.8	6	12.9	15	9.9	5	19.6	11	11.8	14	0.69	B	4
3	France	83.6	11	53.2	13	40.7	15	74.7	11	10.3	13	8.1	8	8.5	2	17.6	9	10.8	15	0.67	B	6
4	Australia	85.9	13	56.9	15	35.9	13	79.5	15	9.4	8	6.4	5	10.4	6	22.8	14.5	15.1	2	0.64	B	7
5	Serbia	94.1	16	62.6	16	40.5	14	80.0	16	10.0	12	13	16	10.8	9	25.4	16	13.8	7	0.85	A	1
6	Czech	82.8	10	50.4	8	42.8	16	73.9	8	10.6	14	8.8	9	10.6	7	21.3	13	12.3	13	0.68	B	5
7	USA	86.5	15	52.3	12	34.9	11	73.7	7	9.3	7	12.8	14	11.8	12	20.6	12	10.5	16	0.74	B	3
8	Poland	77.4	9	52.0	11	31.6	6	76.6	13	6.6	1	5.4	1	9.3	4	17.1	7	12.6	11	0.44	C	9
25	Montenegro	74.0	8	51.9	10	29.7	4	74.2	9	11.6	16	5.8	2	10.8	9	17.2	8	13.2	8.5	0.52	C	8
26	Korea	72.2	7	42.7	2	31.3	5	67.8	1	9.6	10	7.2	7	12.6	14	18.2	10	14.8	4.5	0.42	C	11
27	Angola	70	4	46.6	5	33.7	10	70.7	2	8.6	4.5	6.2	3.5	8.4	1	12.6	2	14.4	6	0.26	D	16
28	Jordan	70.4	5.5	47.1	6	31.7	7.5	72.3	6	9.6	10	6.2	3.5	10.8	9	12.2	1	16.4	1	0.34	D	14
29	Côte d'Ivoire	65.2	1	40.4	1	33.3	9	71.9	3	7.2	3	6.8	6	12.8	15	16.0	6	14.8	4.5	0.34	D	14
30	Senegal	66.0	2	45.8	4	25.9	2	72.1	5	7.0	2	9.6	10	12.0	13	13.4	5	13.2	8.5	0.36	D	12
31	Japan	66.8	3	42.8	3	28.7	3	74.3	10	8.6	4.5	10.2	12	9.0	3	13.0	3	13.0	10	0.36	D	12
32	Philippines	70.4	5.5	47.7	7	25.2	1	72.0	4	9.6	10	10.6	13	13.2	16	13.2	4	15.0	3	0.44	C	9

FR (The final ranking during the 2019 Men's Basketball World Cup), 2P % (2-point field goal made percentage), 3P % (3-point field goal made percentage), FT % (Free throws field goal made percentage), OR (Offensive Rebounds), As (Assists), To (Turnovers), SCP (Second chance points), FBP (Fast-break points).

As can be seen from Table 2, Serbia had the strongest attacking ability, with an RSR value of 0.85, which is the only team belonging to class A level in the top eight; Spain, USA, Argentina, France, the Czech Republic, and Australia all belonged to class B level, with RSR values of 0.76, 0.74, 0.69, 0.67, 0.68, and 0.64, respectively; Montenegro, Poland, the Philippines, and South Korea all belonged to class C level, with RSR values of 0.52, 0.44, 0.44, and 0.42; in turn; Jordan, Japan, Senegal, Côte d'Ivoire, and Angola all belonged to class D level, with RSR values if 0.34, 0.36, 0.36, 0.34, and 0.26, respectively.

### The RSR comprehensive evaluation on defensive performance between the top eight and bottom eight teams

The defensive indicators among the top eight and bottom eight teams were assigned according to the principle of RSR comprehensive evaluation, and finally, the RSR value and grade of each team's defensive performance were calculated. The data shown in Table 3 are the average data for each team. As can be seen from Table 3, the defensive ability of the USA and Spain all belonged to the class A level, with RSR values of 0.81 and 0.80, respectively; Argentina and Serbia all belonged to the class B level, with RSR values of 0.69 and 0.63, respectively; France, Poland, Korea, Montenegro, Senegal, Australia, Czech Republic, Côte d'Ivoire, and Japan all belonged to the class C level, with RSR values of 0.59, 0.56, 0.56, 0.54, 0.53, 0.50, 0.50, 0.50, and 0.48, respectively; Angola, Jordan, and the Philippines belonged to the class D level, with RSR values of 0.29, 0.28, and 0.22, respectively.

**Table 3:** The RSR values and grade of defensive performance between the top eight and bottom eight teams.

**FR**	**Teams**	**LP**	**R**	**DR**	**R**	**Steals**	**R**	**Blocks**	**R**	**Fouls**	**R**	**RSR**	**Grade**	**Rank**
1	Spain	70.0	16	28.5	14	9.0	15	3.3	12	19.4	7	0.80	A	2
2	Argentina	73.9	13	27.6	12	10.0	16	3.0	10	20.8	4	0.69	B	3
3	France	73.4	14.5	25.6	8.5	6.0	4.5	4.3	15	20.5	5	0.59	C	5
4	Australia	81.0	9	29.3	15	4.9	1	2.3	5	18.0	10	0.50	C	10
5	Serbia	74.8	12	28.3	13	7.1	11	2.9	8.5	20.0	6	0.63	B	4
6	Czech	81.4	7	27.5	11	5.0	2	2.5	7	17.4	13	0.50	C	10
7	USA	73.4	14.5	31.3	16	7.5	12	4.0	14	18.4	8.5	0.81	A	1
8	Poland	80.5	10	25.6	8.5	6.3	7	2.9	8.5	17.9	11	0.56	C	6
25	Montenegro	81.2	8	23.8	6.5	8.4	14	3.6	13	21.6	2	0.54	C	8
26	Korea	87.6	4	27.4	10	6.2	6	3.2	11	17.0	14	0.56	C	6
27	Angola	87.0	5	23.8	6.5	5.6	3	2.4	6	21.4	3	0.29	D	14
28	Jordan	96.4	2	21	2	6.0	4.5	1.2	2	17.8	12	0.28	D	15
29	Côte d'Ivoire	80.0	11	20.8	1	6.8	9.5	1.4	3.5	16.0	15	0.50	C	10
30	Senegal	86.4	6	21.6	3	6.8	9.5	4.8	16	18.4	8.5	0.53	C	9
31	Japan	92.8	3	23.4	5	7.8	13	0.6	1	14.0	16	0.48	C	13
32	Philippines	99.8	1	22.4	4	6.4	8	1.4	3.5	22.4	1	0.22	D	16

LP, lost points per game; DR, defensive rebounds.

### The differences in the attacking and defensive performance indicators between the top eight and bottom eight teams

An independent sample *t*-test was conducted to test the differences in the attacking and defensive performance indicators between the top eight and bottom eight teams, including points, 2P%, 3P%, FT%, second chance points, fast-break points, offensive rebounds, assists, turnovers, lose points, defensive rebounds, steals, blocks, and fouls. The data shown in Table 4 are the average data for each team.

**Table 4:** The differences in the attacking and defensive performance indicators between the top eight and bottom eight teams.

	**Attacking performance indicators**									**Defensive performance indicators**				
	**Points**	**2P %**	**3P %**	**FT %**	**SCP**	**FBP**	**OR**	**As**	**To**	**LP**	**DR**	**Steals**	**Blocks**	**Fouls**
Top eight (*x* ± SD)	85.1 ± 4.7	54.2 ± 4.7	36.7 ± 4.2	76.6 ± 2.4	9.5 ± 1.4	9.7 ± 3.0	10.3 ± 1.1	20.9 ± 2.8	12.4 ± 1.5	76.1 ± 4.3	28.0 ± 1.9	7.0 ± 1.8	3.2 ± 0.7	19.1 ± 1.3
Bottom eight (*x* ± SD)	69.4 ± 3.1	45.6 ± 3.6	29.9 ± 3.2	71.9 ± 2.1	9.0 ± 1.5	7.8 ± 2.0	11.2 ± 1.8	14.5 ± 2.3	14.4 ± 1.2	88.9 ± 7.0	23.0 ± 2.1	6.8 ± 0.9	2.3 ± 1.4	18.6 ± 3.0
Difference	15.7	8.6	6.8	4.7	0.5	1.9	−0.9	6.4	−2.0	−12.8	5.0	0.2	0.9	0.5
T	7.945	4.485	3.617	4.186	0.72	1.433	−1.171	5.012	−2.881	−4.435	4.903	0.311	1.462	0.413
*p*-value	0.000^**^	0.001^**^	0.003^**^	0.001^**^	0.484	0.174	0.261	0.000^**^	0.012^*^	0.001^**^	0.000^**^	0.760	0.166	0.686

** *p* < 0.01, with a very significant difference.

* *p* < 0.05, with a significant difference (the same below).

The top eight teams were far better than the bottom eight teams in terms of average points, 2P %, 3P%, FT%, assists, lost points, and defensive rebounds in this Men's Basketball World Cup, and there were very significant differences (*p* < 0.01), while the turnover performance was also better, with a significant difference (*p* < 0.05). In terms of the second attack, fast-break, steals, and blocks, although the top eight teams performed better, there were no significant differences (*p* > 0.05). However, the bottom eight teams were better than the top eight teams in the performance of offensive rebounds and fouls.

### The RSR comprehensive evaluation of the overall attacking and defensive performance between the top eight and bottom eight teams

The overall attacking and defensive performance of a team is a combination of attacking and defensive abilities. To understand the overall competitiveness of each team, it is necessary to conduct RSR comprehensive evaluation and analysis for attacking and defensive performance. The attacking and defensive RSR values of these 16 teams were used as evaluation indexes and assigned according to the principle of RSR comprehensive evaluation. Finally, the RSR value and grade of each team's overall attacking and defensive performance were determined (Table 5).

**Table 5:** The RSR value and grade of the overall attacking and defensive performance between the top eight and bottom eight teams.

**Teams**	**Attack**			**Defense**			**The overall attack and defense**			**Attack-defense characteristics**
	**RSR**	**R**	**Grade**	**RSR**	**R**	**Grade**	**RSR**	**Grade**	**Rank**	
Spain	0.76	15	B	0.80	15	A	0.94	A	1	Both attack and defense strong
Argentina	0.69	13	B	0.69	14	B	0.84	A	4	Both attack and defense strong
France	0.67	11	B	0.59	12	C	0.72	B	5	Attack is stronger than defense
Australia	0.64	10	B	0.50	6	C	0.50	C	10	Attack is stronger than defense
Serbia	0.85	16	A	0.63	13	B	0.91	A	3	Both attack and defense strong
Czech	0.68	12	B	0.50	6	C	0.56	C	6	Attack is stronger than defense
USA	0.74	14	B	0.81	16	A	0.94	A	1	Both attack and defense strong
Poland	0.44	7.5	C	0.56	10.5	C	0.56	C	6	Both attack and defense medium
Montenegro	0.52	9	C	0.54	9	C	0.56	C	6	Both attack and defense medium
Korea	0.42	6	C	0.56	10.5	C	0.52	C	9	Both attack and defense medium
Angola	0.26	1	D	0.29	3	D	0.13	E	16	Both attack and defense weak
Jordan	0.34	2.5	D	0.28	2	D	0.14	E	15	Both attack and defense weak
Côte d'Ivoire	0.34	2.5	D	0.50	6	C	0.27	D	12	Defense is stronger than attack
Senegal	0.36	4.5	D	0.53	8	C	0.39	D	11	Defense is stronger than attack
Japan	0.36	4.5	D	0.48	4	C	0.27	D	12	Defense is stronger than attack
Philippines	0.44	7.5	C	0.22	1	D	0.27	D	12	Attack is stronger than defense

The RSR values of the overall attacking and defensive ability for Spain, USA, Serbia, and Argentina were 0.94, 0.94, 0.91, and 0.84 respectively, which all belonged to class A level; France ranked fifth place, with the RSR value of 0.72, which belonged to class B level. Additionally, Poland, Czech Republic, Montenegro, Korea, and Australia belonged to class C level, with the RSR values of 0.56, 0.56, 0.56, 0.52, and 0.50, respectively; Senegal, Côte d'Ivoire, Japan, and the Philippines all belonged to class D level, with the RSR values of 0.39, 0.27, 0.27, and 0.27, respectively; Angola was the weakest in the overall attacking and defensive ability, with the RSR value of 0.13, and it belonged to class E level like Jordan (0.14). From the perspective of attack and defense characteristics, among the top eight teams, four teams had both strong attack and defense, and three teams had an attack stronger than defense; however, Poland was medium in the attacking and defensive ability.

### The differences in the RSR values of attack-defense ability between the top eight and bottom eight teams

The RSR values of attack, defense, and the overall attacking and defensive performance among the top eight and bottom eight teams were conducted to perform an independent sample *t*-test, to obtain the difference in the attack and defense RSR values. However, the results showed that the bottom eight teams had a large difference in the attack, defense, and overall attacking and defensive abilities, as compared with the top eight teams (*p* < 0.01; Table 6).

**Table 6:** The differences in the RSR values of attack-defense performance between the top eight and bottom eight teams.

	**Attack RSR**	**Defense RSR**	**The overall attacking and defensive RSR**
Top eight (*x* ± SD)	0.7 ± 0.1	0.6 ± 0.1	0.7 ± 0.2
Bottom eight (*x* ± SD)	0.4 ± 0.1	0.4 ± 0.1	0.3 ± 0.1
Difference	0.3	0.2	0.4
T	7.130	3.223	5.164
*p*-value	0.000^**^	0.006^**^	0.000^**^

** *p* < 0.01, with a very significant difference.

### The correlation between the RSR value of attack-defense and the final ranking

**Figure 1:**
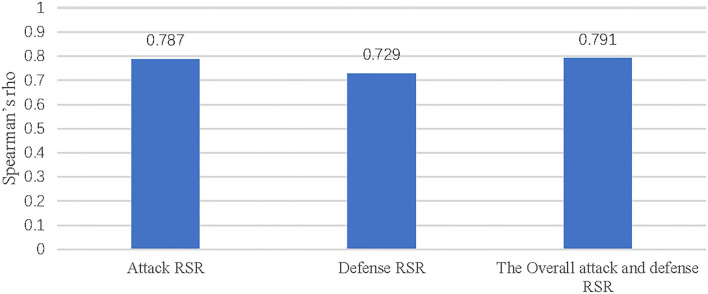
Correlation between the RSR value of attack-defense and the final ranking.

The Spearman Rho correlation analysis was performed at the 0.05 confidence level to test the relationship between the RSR of attack, defense, and the overall attack-defense and the final rankings, respectively. The attack RSR, defense RSR and the overall attack and defense RSR were used as independent variables, and the final rankings of the top eight and bottom eight teams were used as dependent variables, respectively. The correlation coefficients between the independent variables and the dependent variable were obtained in Table 7. At the same time, the data in Table 7 was transformed into Figure 1 in order to more intuitively present the relationship between attack-defense RSR and the final ranking. It can be seen from Table 7 that there was a significant and high relationship between the final ranking and the RSR of attack, defense, and the overall attack and defense (*p* < 0.01), with correlation coefficients of 0.787, 0.729, and 0.791, respectively.

**Table 7:** Correlation between the RSR value of attack-defense and the final ranking.

	**Attack RSR**	**Defense RSR**	**The overall attack and defense RSR**
Spearman's rho	0.787^**^	0.729^**^	0.791^**^
Sig. (2-tailed)	0.000	0.001	0.000
*N*	16	16	16

*Correlation is significant at the 0.05 level (2-tailed).

** Correlation is significant at the 0.01 level (2-tailed).

### The correlation between performance indicators and the attack-defense performance

**Figure 2:**
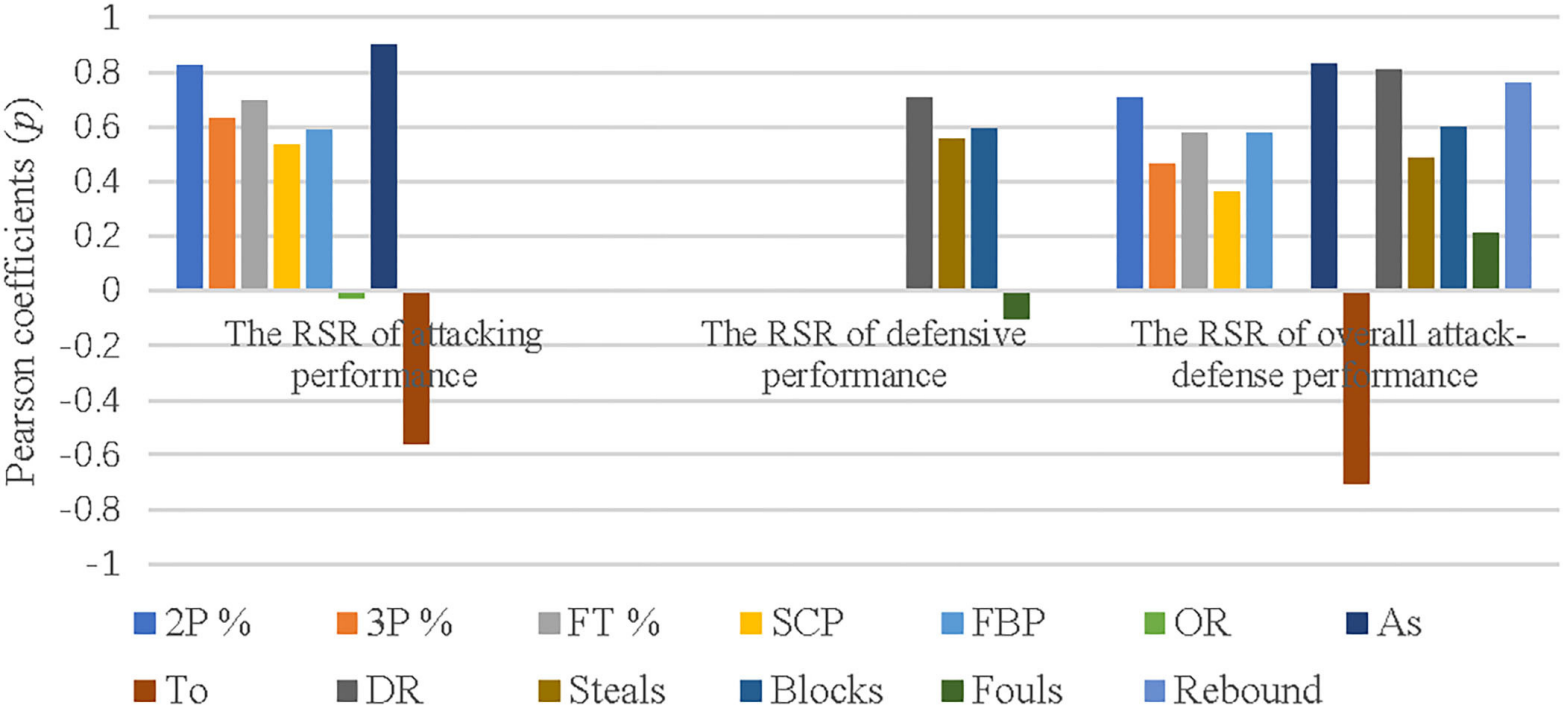
Correlation between performance indicators and the attack-defense performance.

The Pearson correlation analysis was employed to test the relationship between performance indicators and the attack-defense ability at a 0.05 confidence level, which performance indicators of attack and defense as the independent variables, and the RSR of attack, defense, and the overall attack-defense among the top eight and bottom teams as the dependent variables. The correlation coefficients between the independent variables and the dependent variable were obtained in Table 8. At the same time, the data in Table 8 was transformed into Figure 2 in order to more intuitively present the relationship between each performance indicator and the attack-defense performance.

**Table 8:** Correlation between performance indicators and the attack-defense performance.

**Indicators**	**The RSR of attacking performance**		**The RSR of defensive performance**		**The RSR of overall attack-defense performance**	
	**Pearson (*p*)**	**Sig. (2-tailed)**	**Pearson (*p*)**	**Sig. (2-tailed)**	**Pearson (*p*)**	**Sig. (2-tailed)**
2P %	0.827^**^	0.000			0.704^**^	0.002
3P %	0.629^**^	0.009			0.464	0.070
FT %	0.695^**^	0.003			0.575^*^	0.020
SCP	0.532^*^	0.034			0.363	0.167
FBP	0.585^*^	0.017			0.577^*^	0.019
OR	−0.024	0.929			0.000	1.000
As	0.901^**^	0.000			0.832^**^	0.000
To	−0.559^*^	0.024			−0.702^**^	0.002
DR			0.709^**^	0.002	0.809^**^	0.000
Steals			0.555^*^	0.026	0.484	0.058
Blocks			0.594^*^	0.015	0.600^*^	0.014
Fouls			−0.101	0.711	0.213	0.428
Rebound					0.762^**^	0.001

* Correlation is significant at the 0.05 level (2-tailed).

** Correlation is significant at the 0.01 level (2-tailed).

As shown in Table 8, there was a very significant relationship between the attacking performance and assists, 2P %, FT%, and 3P% (*p* < 0.01), with correlation coefficients of 0.901, 0.827, 0.695, and 0.629, respectively. Additionally, the fast-break and second attack had a significant relationship with the attacking performance (*p* < 0.05), with correlation coefficients of 0.585 and 0.532, respectively. In addition, defensive rebounds contributed the most to defensive performance (*p* = 0.709) and had a very significant relationship (*p* < 0.01), at the same time, blocks and steals had a significant relationship with the defensive performance (*p* < 0.05), with the correlation coefficients of 0.594 and 0.555, respectively; Besides that, there was a very significant relationship between the overall attack-defense performance and assists, rebounds, turnovers, defensive rebounds, and 2P % (*p* < 0.01), with the correlation coefficients of 0.832, 0.762, −0.702, 0.809, and 0.704 respectively, while blocks, fast-break points, and FT% had a significant relationship with the overall attack-defense performance (*p* < 0.05). However, 3p%, second chance points, offensive rebounds, steals, and fouls had no significant relationship with the overall attack-defense performance (*p* > 0.05).

## Discussion

According to the principle of the RSR comprehensive evaluation, the attacking and defensive RSR value of the top and bottom eight teams can be obtained by quantifying and synthesizing the specific attacking and defensive indicators. Then the level of attacking and defensive ability among them can be described based on the RSR comprehensive evaluation grade standard, as well as the gap between them. In addition, the disadvantages of the top and bottom eight teams can be described according to the rank and value of performance indicators.

### Attack performance

A basketball competition for both sides to determine the victory or defeat by scores they get within a specified time. Therefore, a strong attacking ability is a basis for a team to get more scores in the game (37). It is worth noting that Serbia's attacking performance was the best, the only team that can reach class A level. However, it placed fifth in this competition, which was inconsistent with its attacking ability. Serbia was defeated by Argentina in the quarter-finals and failed to go further. The statistics after the game showed that Serbia's 3-point shooting rate was only 28.6%, far lower than its average shooting rate (40.5%), while Argentina's was as high as 44.4% (30). Therefore, it seems that the main reason for losing the game was that Serbia had an unstable play, and it did not show its due level in the attacking end. Compared with the last basketball world cup, it was found that the attacking ability of the USA team dropped from A level to B level, because these two indicators free throws and second-chance points which have a significant relationship with attacking performance did not perform well in this competition, while Spain, Serbia, and France remained unchanged, still at the B level (22).

Furthermore, some disadvantages can be found by comparing attacking indicators among the top eight and bottom eight teams. For example, the ability of offensive rebounds for France was insufficient, which can grab 8.5 offensive rebounds per game that was almost the same as that of Angola, and compared with 2-point made percentage (61.8%) ranked first in the last basketball world cup, it has also dropped during this competition (38); Spain's 3-point shooting percentage was low (31.7%), which is the main reason why Spain's attacking ability has not reached the A level, and this disadvantage was actually reflected from the last men's basketball world cup, at only 29.4% (22); Argentina showed poor performance in terms of second attack and offensive rebounds which is the main reason that it cannot reach A level; Australia had too many fouls per game and ranked second to last among 16 teams, and compared with 3-point made percentage (51.9%) ranked first in the last world cup (38), it had dropped significantly in this competition (35.9%); Poland had the worst performance in second attack and fast-break among 16 teams, which were its obvious “short board” that need to improve in the future training. Among the bottom eight teams, Korea's free throw percentage was the lowest which showed the basic skills of players were not good enough. Because this is the only skill without opponent interference, as well as the only skill that all players must perform (38); Angola's offensive rebounds were only 8.4 per game, ranking last, but this indicator for Angola was the best among all teams in the last World Cup (38); Côte d'Ivoire needs to improve 2-point shooting percentage, and the Philippines should strengthen the practice of 3-point shooting, as they reached only 25.2% per game.

### Defense performance

Attack and defense are not only opposite, but also mutually reinforcing during a basketball match, and high-quality defense can provide more attacking opportunities for the team, which is an important guarantee for the team to play well, and also directly determines the outcome of the game (31, 37). The defensive RSR value of the USA was the highest, ranking first, but it only got seventh place in the end, which was inconsistent with its defensive ability. Compared with the last basketball world cup, it was found that the defensive ability of the USA increased from B level to A level, and Serbia's defensive ability increased from D level to B level, however, France and Spain remained unchanged (22).

Furthermore, some disadvantages can be found by comparing defensive indicators among the top eight and bottom eight teams. For example, the Philippines allowed opponents to get 99.8 points per game, which is nearly 30 points more than top-ranked Spain and was the team with the poorest defensive performance, and its fouls also were the most that mean giving opponents too many chances to free throws. Therefore, the Philippines should practice more defensive tactics in future training and be better able to use sound defensive tactics and techniques to reduce fouls; Côte d'Ivoire should strengthen the practice of its ability to grab defensive rebounds, which has a very significant relationship with defensive performance, with only 20.8 defensive rebounds per game, and gave opponents too many chances of the second attack; In terms of steals, the fourth-ranked Australian was the worst, with only 4.9 steals per game, and Czech had 5.0 steals per game, both of them did not make big defense stress to their opponents, which might be the important reasons of the insufficient defensive ability for them. Japan had the weakest ability on blocks, with only 0.6 per game.

### The overall attacking and defensive performance

In basketball, a single attacking or defensive ability only reflects a team's unilateral performance, while the combination of attacking and defensive ability is the embodiment of a team's overall strength (39). The overall attacking and defensive ability of the USA belonged to the class A level. However, the USA only got seventh place. The USA lost to France in the quarter-finals, which made it unable to go further. According to the post-game technical statistics, the main reasons for the USA team's loss were that rebounds were far below their average level, with offensive rebounds (9), defensive rebounds (19), and total rebounds (28). On the contrary, the French played well in rebounds, which offensive rebounds (13), defensive rebounds (31), and total rebounds (44) being higher than their average level, with 16 rebounds more than the USA (30). Therefore, stable performance in key matches was also crucial for the team, especially for teams with close strength. Compared with the previous Men's Basketball World Cup, Spain's overall attacking and defensive ability increased from B level to A level, and Serbia also increased from C level to A level, however, USA and France did not change (22).

The top basketball teams, especially those which reached class A level in the overall attacking and defensive ability, generally have the characteristics of strong attack and defense, and they have focused on developing both attack and defense, it is worth learning from other countries. This result also has been reported by previous studies (39, 40). Unfortunately, some teams with strong overall attacking and defensive abilities failed to get better results, such as the USA and Serbia. However, there is only one champion, and the main reason for this situation is that the two teams are not stable enough in key competitions. Besides that, in competitive sports, no team can always win due to the inherently unpredictable nature of sports, and the numerous potential factors that can affect results. This unpredictable nature is a major part of the charm of competitive sports, and also one of the main reasons that people enjoy sports (41). But it is undeniable that these two teams will still be strong competitors for the championship from the perspective of overall strength in the next few years.

### The correlation between attacking and defensive performance and the final ranking

This study showed that there was a very significant and high correlation between the final ranking and attack, defense, and overall attack-defensive ability, just the degree of correlation was different among them. Compared with previous studies, this result is consistent with the results reported by Kang and Yuan (24), and Li et al. (25). However, the results of some studies differ from this study. For instance, Li and Sun (21) stated that there was a significant relationship between the RSR value of defense and the final ranking during the 2015 Asian Men's Basketball Championship, while the attacking RSR value was not highly relevant to the final ranking. Besides that, a strong correlation between attack and the final ranking has been found in other studies (22, 23). Additionally, according to the correlation coefficient between the RSR value of attack and defense and the final ranking. The win or defeat was largely determined by the performance of both sides of the overall attack and defense during the 31st Rio Olympic Games (31). Other researchers also got a similar result. For instance, Zhu and Yu (39) stated that the balance of attack and defense was the key factor to getting good game outcomes among college students in men's basketball games. In short, combined with the common attack-defense characteristics of the top teams, this study shows that attack and defense are indispensable to team performance. Both need to be promoted to improve the team's strength and achieve good results. Coaches should focus on this view during training in the future.

### The correlation between the overall attack-defense performance and performance indicators

As we all know, basketball is a team game including defense and attack and requires various types of interaction (42). In the 2019 Men's Basketball World Cup, assists contributed the most to the top eight teams, followed by rebounds, turnovers, and 2P%, both of them had a very significant and high relationship with the overall attacking and defensive ability. According to this result, the team that has the more assists and rebounds, the higher 2P%, and the fewer turnovers, the final ranking is higher.

Assists can be used to measure teamwork and require good decision-making on the court, as they reflect the cooperation ability among teammates (43). Similar results were found by comparing previous studies. For example, by using the discriminant analysis method, it was found that assists were quite an important indicator for the winning teams (6, 14, 44, 45). In addition, Gómez et al. (46) examined women's basketball competitions and demonstrated that assists were an important indicator of match success. However, one study indicated that assists did not contribute to distinguishing high-ranking teams in the first Croatian basketball league, which showed different results from this study (47). Additionally, rebounding dominance is seen as a key element of the basketball game for the best teams (16, 48, 49), and it is the beginning of an attack action, especially for the second attack and fast-break. There were some similarities and differences by comparing with previous studies. Lots of previous research declared that the rebound was clearly a very important variable to discriminant the victory and defeat in the basketball game (1, 10, 14), especially defensive rebounds (10, 16, 18, 19, 40, 50, 51), and some studies also showed that offensive rebounds were more important in determining game results (52). However, the result of this study stated that the offensive rebounds had no correlation with the overall attacking and defensive performance. This means that the bottom teams also have a strong offensive rebounding ability, and there is no gap between the top and bottom teams, and even better than the top teams in this study. In addition, field goal percentage (50, 52), turnovers (14, 52), and free throws (52) have also been identified as important factors in distinguishing the best teams, and this study also showed the same results, except for 3P%.

Fast-break played an increasingly important role in the game as the pace of basketball games got faster and faster (53), and a large number of fast-break led offensive players easier to get scores (6, 44). For example, some studies showed that fast breaks might be one of the main indicators differentiating between winning and losing teams in both women's and men's basketball (54), and this study also agrees with this view, which showed a significant correlation between fast-break and the overall attacking and defensive ability. Furthermore, Dogan et al. (14) have indicated that steals may be an important indicator to distinguish the winning and losing teams. Steals are usually the reason for the team to launch the fast breaks in basketball. However, the results of this study showed that the overall attacking and defensive performance had no significant relationship with steals. Because it is possible that a steal results in a fast break but does not necessarily result in a final score, there is no decisive relationship between steals and fast-break scores. Therefore, the team should improve its ability to effectively convert steals into fast-break points, which is also an important point for the coach to think about. Although the number of second attacks is increasing in basketball competition with the constant changes in rules (33), previous studies did not show a quantitative relationship between second attack and attacking performance, while this study has indicated that the second attack is not a significant factor to discriminate between the top and bottom teams. Moreover, this study found that blocks had a significant relationship with overall attacking and defensive ability, but no similar results were found in previous studies.

As shown by the discussion above, there are many differences among studies concerning the contribution of performance indicators and attack-defense ability to game outcomes. Analyzing the reasons that may be because the studies were carried out from different levels, nations, or nature competitions utilizing different analysis methods. Additionally, the continuous development of basketball and changes in rules are possible reasons for the inconsistency of the research results.

## Limitation

A major limitation of this study was that it only analyzed the 2019 Men's Basketball World Cup to determine the attacking and defensive ability of each team. Some teams may not play their real strength for various reasons in this competition. Therefore, the analysis of multiple high-level competitions can fully reflect the attacking and defensive ability of the team in future studies. The tactics decision by the coach during the game, such as outside or inside attacking, will also have an impact on the game's statistics to a certain extent. In addition, basketball is a complex team sport where the game events and situations are dynamically related across the game (55). During the past years, several studies examined the effects of situational variables such as game location, match status, and the quality of the opponent on performance indicators (56). However, this study did not control for the influence of situational variables on attacking and defensive performance, which is also one of the limitations. Differences in essential characteristics of players between the top and bottom teams, such as physical and physiological characteristics, may also be one factor that causes the teams' different attacking and defensive performances. Although, this aspect was not considered in this study, and a more comprehensive study is expected to be conducted in the future.

## Conclusion

The result of this study showed that the top basketball teams focused on developing both attack and defense, and have the common characteristics of strong attack and defense. Whether it was the attack, defense, or overall attacking and defensive ability, there was a significant relationship with the final ranking. Additionally, this study showed that there were very significant differences in both attacking and defensive abilities between the top eight and bottom eight teams, as well as highlighted their respective advantages and disadvantages in attacking and defensive indicators. Besides that, this study found that performance indicators such as assists, defensive rebounds, 2P%, turnovers, FT%, fast-breaks, and blocks were the main factors that distinguish the top and bottom teams, and they had a significant relationship with overall attacking and defensive performance. The above information allows coaches and players to learn the latest developments in competitive basketball, as well as their advantages and disadvantages, to help them organize targeted training in the future.

## Data availability statement

The original contributions presented in the study are included in the article/supplementary files, further inquiries can be directed to the corresponding author/s.

## Author contributions

WS was responsible for the conception and writing of the article. CC was responsible for the supervision and editing of the article. LK was responsible for the revision of the article. FL was responsible for the statistical analysis of the article. SS was responsible for the data collection of the article. All authors contributed to the article and approved the submitted version.

## Conflict of interest

The authors declare that the research was conducted in the absence of any commercial or financial relationships that could be construed as a potential conflict of interest.

## Publisher's note

All claims expressed in this article are solely those of the authors and do not necessarily represent those of their affiliated organizations, or those of the publisher, the editors and the reviewers. Any product that may be evaluated in this article, or claim that may be made by its manufacturer, is not guaranteed or endorsed by the publisher.
